# Effect of Amino Acid Substitutions on Biological Activity of Antimicrobial Peptide: Design, Recombinant Production, and Biological Activity

**DOI:** 10.22037/ijpr.2019.112397.13734

**Published:** 2019

**Authors:** Parvaneh Panahi Chegini, Iraj Nikokar, Maryam Tabarzad, Sobhan Faezi, Arash Mahboubi

**Affiliations:** a *Department of Medicinal Biotechnology, School of Paramedicine, Guilan University of Medical Sciences, Rasht, Iran. *; b *Medical Biotechnology Research Center, School of Paramedicine, Guilan University of Medical Sciences, Rasht, Iran. *; c *Protein Technology Research Center, Shahid Beheshti University of Medical Sciences, Tehran, Iran. *; d *Department of Pharmaceutics, School of Pharmacy, Shahid Beheshti University of Medical Sciences, Tehran, Iran.*

**Keywords:** Antimicrobial peptide, Chitin binding domain, Intein linker, Recombinant production, Amino acid substitution

## Abstract

Recently, antimicrobial peptides have been introduced as potent antibiotics with a wide range of antimicrobial activities. They have also exhibited other biological activities, including anti-inflammatory, growth stimulating, and anti-cancer activities. In this study, an analog of Magainin II was designed and produced as a recombinant fusion protein. The designed sequence contained 24 amino acid residues (P24), in which Lys, His, Ser residues were substituted with Arg and also, hydrophobic Phe was replaced with Trp. Recombinant production of P24 in *Escherichia coli (E. coli)* BL21 using pTYB21, containing chitin binding domain and intein sequence at the N-terminus of the peptide gene, resulted in 1 μg mL^-1^ product from culture. Chitin column chromatography, followed by online peptide cleavage with thiol reducing agent was applied to purify the peptide. Antimicrobial activity was evaluated using five bacteria strains including *Staphylococcus aureus, Enterococcus faecalis, Klebsiella pneumonia, E. coli, *and* Pseudomonas aeruginosa*. Designed AMP exhibited promising antimicrobial activities with low minimum inhibitory concentration, in the range of 64-256 µg/mL. P24 showed potent antimicrobial activity preferably against Gram-positive bacteria, and more potent than pexiganan as a successful Magainin II analog for topical infections. In general, further modification can be applied to improve its therapeutic index.

## Introduction

One of the main challenges in global health services is the emergence of antibiotic-resistant infections. Various strategies have been evaluated regarding the treatment of resistant-infections including improved drug delivery systems (1-3). Moreover, introducing potent and broad-spectrum antimicrobial agents have been considered as an important healthcare success. Anti-microbial peptides (AMPs) are short length peptides which exhibit potent and wide spectrum antimicrobial activity. They can be used against antibiotic-resistant pathogens, such as methicillin-resistant *Staphylococcus aureus* (MRSA), vancomycin-resistant *Enterococci* (VRE), multi-drug-resistant *Pseudomonas aeruginosa*, and *Mycobacterium tuberculosis *(4). Most of the AMPs have positive charges and alpha helix structure with amphipathic conformation that can disrupt microbial membrane (5). Although positive charge and the presence of hydrophobic residues are the common features of AMPs; however, a wide diversity in other physicochemical properties of AMPs, including sequence of amino-acids, size, and structure have been reported (6). More than the AMPs’ effect on membrane integrity, they potentially act as potent antibiotics through making an alteration in microbial metabolic pathways or inhibit DNA, RNA, protein, and cell wall synthesis (7). 

AMPs have been extracted from diverse natural sources, particularly from plants, insects, animals or human. In addition, they could be synthesized by chemical process, as well as production by recombinant technology (8, 9). Chemical synthesis and extraction from natural sources are not cost-effective approaches for large-scale production of antimicrobial peptides. Accordingly, recombinant DNA technology has been extensively studied to economically produce AMPs in large scale. In fact, AMPs are toxic for bacterial expression systems and due to the small size and cationic characteristics, they are prone to proteolytic degradation. Therefore, the fusion of AMP to a carrier protein has been considered to overcome these issues in their recombinant production (10, 11). One of the fusion tags for efficient and simple recombinant production and purification is the chitin-binding domain (CBD), which has also been used for the production of AMPs in *Escherichia coli (E. coli)* expression system (12). Using this system (13, 14) results in a simple protein purification steps, since the high-affinity and high-specificity interaction between the CBD and chitin provides a simple strategy for efficient protein or peptide purification. Moreover, the presence of the intein cleavage system simplified the elution of protein with a chemical reagent, without the need for a specific protease to remove the affinity tag (14). 

One of the promising strategies for simple purification of an untagged protein is the fusion of a chitin-binding domain (CBD) followed by a self-cleaving intein sequence, which allows one-step chromatographic purification of desired protein using the thiol-catalyzed cleavage of the intein sequence (14). The chitin binding tag, commonly used are originated from *Bacillus circulans* and consists of 51 amino acid residues. The recombinant form of this protein and their fusion partners can bind to the chitin sorbent like chitin immobilized on Sepharose, under physiological conditions. Elution of these proteins from sorbent requires severe conditions, including high concentrations of alkali, which would be harmful for the desired product. Therefore, CBD tag is frequently used in a complex with intein as an easily self-cleavage polypeptide (15). Some modifications should be applied to intein self-cleavage system to make it suitable for protein purification in a way that the cleavage of inteins is turned off *in-vivo* and later activated *in-vitro*, for example by in traducing a mutation at the intein C-terminal Asp to Ala (16). This type of recombinant production system including CBD and intein fusion tag for AMPs production have been reported for cecropin (17), dermcidin (18), and some other AMPs (19, 20).

One of the typical antimicrobial peptides is magainin II, which was extracted from the skin of African clawed frog, *Xenopus laevis*, and its potent and broad-spectrum antimicrobial and antitumor activities have been confirmed. Numerous derivatives of magainin II have been introduced with improved antimicrobial activities or the emergence of other pharmacological features (21). In this study, a novel AMP was designed based on the sequences and structures of magainin II and its analog, Pexiganan, and then, was produced as a recombinant fusion polypeptide containing chitin-binding domain at N-terminal, intein linker at C-terminal. Functional activities of the designed AMP were evaluated by antibacterial activity and hemolytic effect. 

## Experimental

*Chemicals*

Polymyxin B sulfate salt, Ampicillin, Tetra-cycline, Acrylamide/bisacrylamide solution (30%), IPTG and Protein ladder were supplied from Sigma (USA). Other chemicals were purchased from Merck Chemicals (Germany). SYBR green (Safe stain) and 50 bp DNA ladder were supplied from Sinaclon (Iran). NdeI was purchased from Takara (Japan) and EcoRI from Thermo Scientific (USA).

*Peptide design as a novel AMP*

To design a new AMP, Magainin II was considered as the template AMP with promising activity against a broad microbial agent. Peptide sequences of magainin II and pexiganan was extracted from the antimicrobial peptide Database (APD; http://aps.unmc.edu/AP) (22). Magainin II sequence is GIGKFLHSAKKFGKAFVGEIMNS. The peptide length was shortened and then, Lys, His, and Ser residues from magainin II sequence were substituted with Arg and also, hydrophobic Phe with Trp. Various peptide sequences were designed and analyzed by dPABBs (Design Peptides against Bacterial Biofilms) web server (http://ab-openlab.csir.res.in/abp/antibiofilm/index.php) (23). The best designed peptide (P19) had 19 amino acid residues. Then, according to the structure of the vector pTYB21, some modifications were applied in to the designed AMP (P19) and five amino acid residues (GRAHM) were added to the N-terminal of P19, which resulted in 24 amino acid sequence of P24. Physicochemical characteristics of the designed peptides were predicted by PepFold (http://bioserv.rpbs.univ-paris-diderot.fr/services/PEP-FOLD/) and Helical Wheel draw program from Raphael Zidovetzki’s lab (http://rzlab.ucr.edu/scripts/). 

*Peptide and DNA sequences*

**Figure 1:**
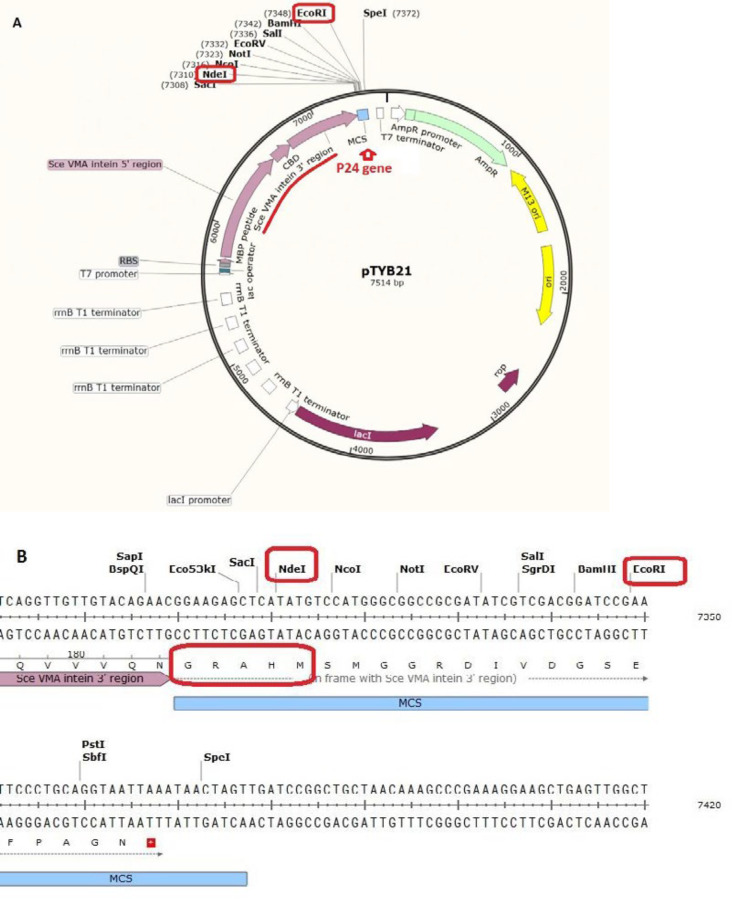
The structure of recombinant vector pTYB21 for novel designed AMP (P24). (A) The structure of pTYB21; the important parts are outlined with red box. (B) The sequence of first 5 amino acids at MCS of pTYB21, was added to the N-terminal of P19, indicated in red box

Pexiganan (GIGKFLKKAKKFGKAFVK-ILKK-NH_2_) was chemically synthesized by Dr. Kobarfard group (School of Pharmacy, Shahid Beheshti University of Medical Sciences, Tehran, Iran). Vector pTYB21 (cat# N6709S, Figure 1) was supplied from New England Biolab company (UK). The vector contains chitin binding domain and Sec VMA intein sequence upstream of the multiple cloning site (MCS). pTYB21 had ampicillin resistance gene as the selection marker. Universal primers of this vector were synthesized by Sinaclon Company (Iran). After designing the peptide, the P24 AMP gene was reverse-translated from amino acid sequence using the EXPASY bioinformatics tool (Reverse translate tool) and the codons were optimized according to the *E. coli* expression system. Gene plus (5´-TTTTTTT CAT ATG CGC TGG TTA CGC CGT TGG CGT CGC TGG GGC CGC GCC TGG GTC CGT ATT CTT CGC CGC TAA GAA TTC TTTTTTT- 3´) and gene minus (5´- AAAAAAA GAA TTC TTA GCG GCG AAG AAT ACG GAC CCA GGC GCG GCC CCA GCG ACG CCA ACG GCG TAA CCA GCG CAT ATG AAAAAAA- 3´) were synthesized by Bioneer (Korea). The P24 gene was designed with NdeI restriction site at 5’ end and EcoRI restriction site at the 3´end of the gene for directed cloning into the pTYB21. A stop codon was designed at the 3´end of the gene. 

*Cloning and transformation of AMP gene-containing construct*

The P24 gene was digested with NdeI at 5’ end and EcoRI at the 3´end of the gene for site-directed cloning into the pTYB21. After confirmation of the desired recombination by polymerase chain reaction (PCR) and electrophoresis on agarose gel, the recombinant vector was cloned into the *E. coli *TOP10F’ that had an endogenous plasmid with tetracycline resistance gene as the selection marker). The positive clone, which grow on ampicillin and tetracycline supplemented LB-agar culture plate, was selected and the recombinant plasmid was extracted and transformed into the *E. coli* BL21 for protein expression using calcium chloride heat-shock method. 

The fusion gene composed of the designed peptide and the intein-chitin binding domain is under the control of T7 promoter, which can be readily induced by isopropyl b-D-thiogalactoside (IPTG). From the overnight culture of *E. coli* BL21, cell harboring recombinant pTYB 21 constructs was sub-cultured (1:100) in fresh LB broth medium supplemented with 0.1 mg mL^-1^ ampicillin and grown at 37 °C under agitation. Expression of the fusion protein was induced by the addition of IPTG to a final concentration of 400 µmol L^-1^ (when the OD_600nm_ of culture reached to 0.8). After induction, the cultures were incubated at 37 °C. To find the best time of cell harvesting, the 1 mL sample of culture was harvested every hour until 5 h (19).

*CBD-AMP fusion protein production in E. coli*

A suspension (500 mL) of recombinant *E. coli* BL21 in LB broth medium was cultured at 37 °C. Four hours after IPTG induction, the cells were harvested by centrifugation at 4500 ×g, at 4 °C for 15 min and washed three times with PBS. The supernatant was removed and evaluated by SDS-PAGE 15%. The bacterial pellet was re-suspended in buffer-1 (20 mmol L^-1^ Tris–HCl, pH 8.0, 0.1 mmol L^-1^ EDTA, 0.5 mol L^-1^ NaCl, 20 µmol L^-1^ PMSF) and disrupted by sonication (200 W power for 25 min in 30 sec intervals of off/on program) (19). Lysis mixture was centrifuged at 15000 ×g, at 4 °C for 30 min. The release of protein was monitored using the Bradford assay. The pellet and supernatant were separated and checked for the presence of the fusion protein by SDS-PAGE 15%. 

*Purification of the desired AMP from inclusion bodies*

Pellet of inclusion bodies was solubilized in buffer-2 (urea 8M in column buffer). The mixture was vortex and centrifuged at 12000 ×g for 20 min at 4 °C. The supernatant, containing fusion protein (56 kDa), was dialyzed against buffer-1 with gradient reduction of urea concentration using 12 kDa dialysis tube (#D0405, Sigma, USA) at 37 °C to refold the protein. The resulted solution was loaded onto a 10 mL chitin column and washed with 10-bed volumes of buffer-1, then, the column was flushed quickly with 3-bed volumes of buffer-3 (elution buffer: 20 mmol L^-1^ Tris–HCl buffer (pH 8.0), 0.1 mmol L^-1^ EDTA and 0.5 mol L^-1^ NaCl containing 50 mmol L^-1^ DTT). The column was incubated at room temperature for 40 h to induce self-cleavage of the fusion protein. After that, the protein was eluted from the column by 2-bed volumes of elution buffer. The cleavage efficiency was determined using SDS-PAGE 15%.

*Antimicrobial activity assay*

Antibacterial activity assay was performed by microdilution method (24). Briefly, 0.1 mL of Muller-Hinton broth medium, containing CaCl_2 _and MgCl_2 _(final concentration of calcium 20 µg mL^-1^ and magnesium 10 µg mL^-1^), was added to each well of a 96-well microtiter plate. The primary stock of P24 peptide was provided to 10 mg mL^-1^ concentration. Serial dilution of the P24 peptide and pexiganan in the range of 4-512 µg mL^-1^ was prepared in separate wells in triple sets. 0.01 mL of the 1:20 dilution of 0.5 McFarland suspension (1 × 10^8^ CFU mL^-1^), which contains 5 × 10^6^ CFU mL^-1^ was added to each well. Bacterial suspensions included *Pseudomonas aeruginosa (P*. *aeru*g*inosa)*, *Staphylococcus aureus* (*S. aureus)*, *Enterococcus faecalis (E. faecalis), Escherichia coli (E. coli), Klebsiella pneumoniae (K. pneumoniae)*. The plates were incubated at 35 ± 2 °C and ambient air for 24 h before reading the MICs.

*Hemolytic activity assay*

Hemolytic activity was assayed as described by Jang *et al.* (25). Briefly, 3 mL of freshly prepared human red blood cells (RBCs) was washed with isotonic PBS (pH 7.4), until the color of the supernatant became clear. The washed RBCs were then suspended in the final volume of 20 mL using BPS, pH 7.4. Ten microliters of peptide samples, serially diluted in PBS (final concentrations ranging from 0.15 to 150 µg mL^-1^), was added to 190 mL of the cell suspension in microfuge tubes. Following gentle mixing, the tubes were incubated at 37 °C for 30 min and then, centrifuged at 4000 ×g for 5 min. Finally, 100 mL of supernatant was diluted to 1 mL with PBS and then, absorbance at 567 nm was measured for monitoring the release of hemoglobin. The release of hemoglobin indicated RBC membrane damage. Negative control with no hemolysis and the positive control with 100% hemolysis were determined in PBS and 0.2% Triton X-100, respectively. The percentage of hemolysis was calculated using the following Equation: $\mathrm{Haemolysis}\left(\%\right)=\frac{\left(A_{s}-A_{0}\right)}{\left(A_{100}-A_{0}\right)}\times 100$ , where A_s_ is the absorbance of the sample, A100 is the absorbance of completely lysed RBCs in 0.2% Triton X-100 and A_0_ is the absorbance in the complete absence of haemolysis.

## Results

*Designing of novel AMP*

**Figure 2:**
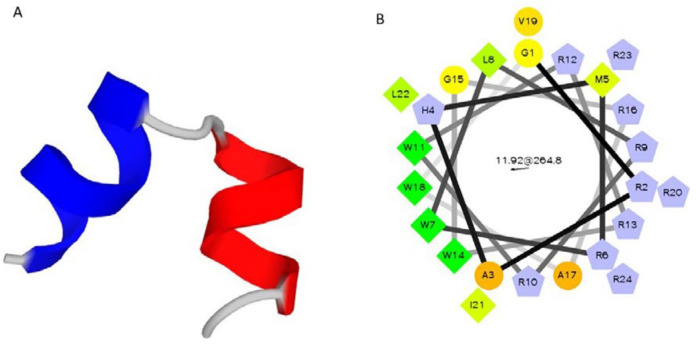
(A) Predicted structure of designed peptide P24 by PepFold ((http://bioserv.rpbs.univ-paris-diderot.fr/services/PEP-FOLD/) with two distinct helical structures; (B) Helical Wheel structure of designed peptide P24 with obvious amphipaticity; green: most hydrophobicity, yellow color: least hydrophobicity, circle: hydrophil amino acids, pentagonal: positive charge amino acids, triangle: negative charge amino acids. Evaluated by Helical Wheel Projections from RZ Lab (http://rzlab.ucr.edu/scripts/ wheel/wheel.cgi?submit)

In the present study, Magainin II and its analogs sequences, especially Pexiganan, have been considered for novel peptide design. Peptide sequences of magainin II and pexiganan were extracted from the antimicrobial peptide Database (22). There are also several AMPs that were well designed based on Arginine (Arg), as positive hydrophilic residue, and Tryptophan (Trp), as hydrophobic residue in peptide primary structure. These designed AMPs, including WR 12, PB100, or RW-BP100, exhibited promising antimicrobial activity and therapeutic index (5, 26 and 27). Therefore, Arg and Trp can be effective substitutions to develop novel successful potent AMPs. Regarding these reports, a sequence containing 19 amino acid residues (RWLRRWRRWGRAWVRILRR) was designed that in this peptide (P-19), Lys, His, Ser residues from magainin II sequence were substituted with Arg and also, hydrophobic Phe with Trp. Prediction of biological activities of the new antimicrobial peptide sequence, using dPABBs (Design Peptides against Bacterial Biofilms) web server (http://ab-openlab.csir.res.in/abp/antibiofilm/index.php) (23) confirmed the anti-biofilm activity of P-19. In addition to the biological activity, PepFold (28) was used to provide the structural and conformational information. Previously, Zasloff *et al.* illustrated that omission of three amino acids from N-terminal of magainin II did not affect peptide function (29). Moreover, 12 amino acids peptide (RW12) was designed that only contains arginine and tryptophan residues with optimal amphipathicity, alpha helix structure and good antimicrobial activity on a broad spectrum of gram negative and gram positive bacteria (30). According to these findings and considering the vector pTYB21 sequence for recombinant expression of the peptide, in this study, a novel AMP (P24) was designed, which consisted of 10 Lys and 4 Trp residues. Five amino acids of GRAHM were added to the N-terminal of the designed peptide to adopt with the pTYB21 reading frame, resulted in 24 amino acids’ sequence (P24) of GRAHMRWLRRWRRWGRAWVRILRR. P-24 demonstrated a similar predicted structure and bioactivity using PepFold and dPABBs tools. Physicochemical properties of P24 were reported in Table 1. The helical structure of a designed peptide (P-24) was predicted by PepFold (Figure 2A). As the alpha-helical structure of cationic AMPs and their amphipathicity are two important features of these peptides, alpha-helix formation and the positions of hydrophobic and hydrophilic residues of P24 were evaluated by Helical Wheel Projections from RZ Lab (Figure 2B). The helical wheel is a type of visual representation to illustrate the characteristics of alpha helices in peptides and proteins (31, 32). Helical wheel illustration of peptide confirmed its amphipathicity. 

*Recombinant gene design for novel AMP*

**Figure 3:**
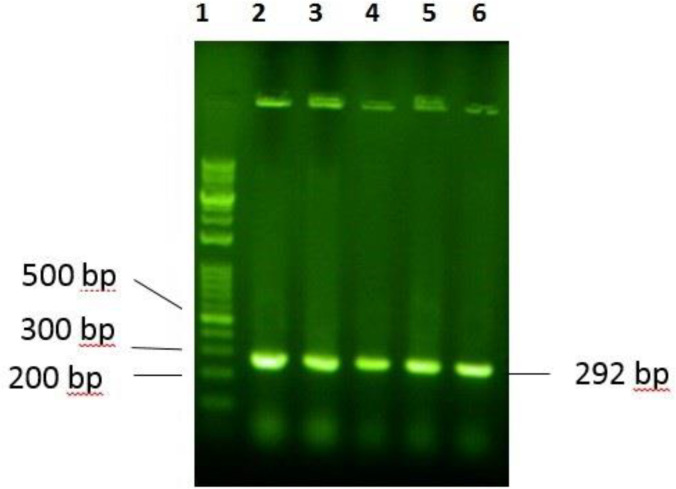
Electrophoresis evaluation of PCR products on agarose gel 2%: 1) ladder 100bp-10 kbp 2) PCR products of selected colons

Gene sequence adopted from the amino acid sequence of P-24 was chemically synthesized and then, ligated to the pTYB21, using NdeI and EcoRI restriction sites. DNA digestion and ligation were primarily confirmed by agarose 2% gel electrophoresis. Transformed *E. coli* TOP10 F´ were analyzed by colony PCR, using universal primers and DNA sequencing. The 292 bp DNA bond indicated the presence of the favored gene in the multiple cloning sites of the vector (Figure 3). The plasmid of positive colons was extracted and sequenced. Result of sequencing confirmed the presence of the desired gene in the recombinant construct. The recombinant plasmid was transformed into competent cells of *E. coli *BL21 as the expression host.

*Recombinant production and purification of designed AMP*

**Figure 4:**
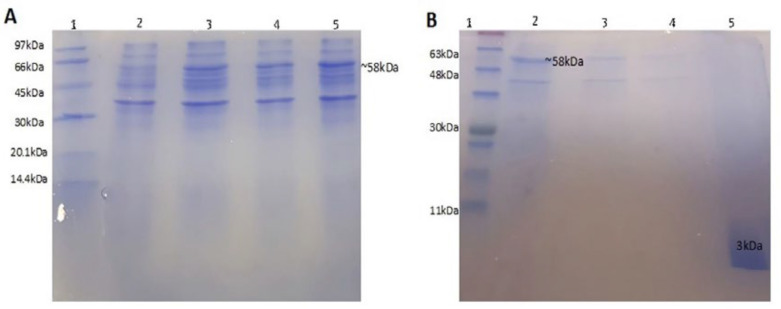
Electrophoretic analysis on 15% SDS–PAGE (A) of intein-fusion expression in *E. coli* BL21. Lane 1, Low molecular weight protein ladder; Lane 2, before IPTG induction (un-induced); Lane 3, 1 h after IPTG induction; Lane 4, 3 h after induction; Lane 5, 4 h after induction; (B) samples obtained from different steps of purification. Lane 1: protein ladder, lane 2: sample after solubilization of inclusion bodies loaded on chitin column, lane 3: samples after first wash, lane 4: sample after second wash, lane 5: purified peptide after cleavage by DTT

Transformed colons were selected and cultured in LB broth. After IPTG induction, fusion protein production was evaluated during 5 h culture, every 1 h, using SDS-PAGE 15%. Results showed that the production reached the maximum value after 4 h (Figure 4A). In this study, a one-step protein purification system was used to facilitate purification of the peptide. This system uses self-catalyzed cleavage of the intein that is induced by DTT (1,4-dithiothreitol). The chitin-binding domain (CBD) served as an affinity tag to bind intein-peptide fusion onto a chitin column. The recombinant peptide was released from the fusion after the induction of the intein cleavage reaction by addition of 50 mM DTT, while intein-CBD fusion remained bound to the column (33, 34). Using urea 8M as solubilizing agent resulted in the best recovery of the fusion protein form the inclusion bodies. After refolding, the fusion protein was extracted from other concomitant proteins by capturing on chitin column. P-24 was eluted from the column by adding DTT, as a thiol containing reducing agent cleaved the intein linker and released the peptide from the fusion protein. Resulted peptide was concentrated and evaluated by SDS-PAGE 15% (Figure 4B). The band of designed peptide with the molecular weight of around 3 kDa was observed under the ladder band of 11 kDa. Purified protein concentration was calculated based on the absorbance in 280 nm and it extinction coefficient (22760 M-1 cm-1) that resulted in about 1 mg/L production yield. 

*Antimicrobial activity of novel AMP*

Antibacterial activity of P-24 was assessed against both gram-negative and gram-positive bacteria (Table 2). The best results were observed against *E. faecalis and S. aureus *and the reported MIC was 64 µg/mL that confirmed the superior activity of P24 on gram-positive bacteria compared to pexiganan. It exhibited promising anti-microbial activity against *P*. *aeruginosa, *which was similar to the pexiganan anti-microbial activity. The reported MICs against gram negative bacteria including *E. coli *and* K. pneumonia* confirmed that novel AMP can be considered as a more potent AMP than pexiganan (128 µg/mL compared to 512 µg/mL, respectively).

*Hemolytic activity of novel AMP*

Hemolytic activity of the P24 was studied compared to pexiganan. The results indicated that the hemolytic activity started to form about 1 µg mL^-1^ and reached to about 26% at 150 µg/mL. However, Pexiganan did not show significant hemolysis compared to PBS at the same concentration. 

## Discussion

Nowadays, resistant infections are one of the important challenges of world health. Therefore, the design and development of new antibiotics, which can be active against resistant pathogens is an interesting field of medicine. An interesting group of antimicrobial agents with potent activity against resistant bacteria and also, fungus and viruses are antimicrobial peptides (AMPs) (35). Designing the novel AMPs has been commonly performed based on the physicochemical parameters including net charge, hydrophobicity, hydrophobic moment, and polar angle (36), as well as, α- helix structure and amphiphilicity, which are highly impressive on peptide activities. AMPs typically influence the bilayer- lipid membrane of bacteria to exert their antimicrobial activity (37). Studies indicated that arginine and lysine substitution might let to primarily facilitated insertion into bacteria membrane. Also, it was indicated that the setting of hydrophobic amino acids such as tryptophan and phenylalanine in peptide sequence intensified antimicrobial activity against bacteria (38). In this study, a 19 amino acid peptide (RWLRRWRRWGRAWVRILRR) was first designed (P19), in which, Lys, His, Ser residues from magainin II sequence were substituted with Arg and also, hydrophobic Phe with Trp. Then, five amino acids of GRAHM were added to the N-terminal of the designed peptide to adopt with an expression vector, resulting in a 24 amino acids sequence (P24) of GRAHMRWLRRWRRWGRAWVRILRR. P24 demonstrated similar alpha-helical structure and bioactivity using bioinformatics tools. According to the relatively similar helical wheel structures drawn for Pexiganan, P19, and P24, therefore, the designed peptides had good amphipathicity as an AMP. 

Besides, peptides more than 10 amino acids are more cost-effective to be produced by recombinant production instead of chemical synthesis. Recombinant production of peptides was commonly performed in the structure of a fusion protein, which can improve the production and purification of the desired peptide, as well as decrease cytotoxicity, especially in the case of AMPs (39). After recombinant production, the separation of fusion parts from the desired peptide or protein is commonly done by digestion with endoproteases that is an expensive approach. Several chemicals have also been used to facilitate the cleavage of special sequences like intein. Inteins with self-cleavage ability are interesting linkers in the recombinant production of peptides (40, 41). In this study, pTYB21 was considered in the recombinant production of P24. pTYB21 contains intein linker joined to a chitin-binding domain (CBD), which facilitated peptide purification by chitin column (42). Similar reports have successfully used this strategy for effective production of AMPs in bacterial hosts (17, 20 and 33). P24 started with Gly, Arg, Alanine, His, and Met residues at the N-terminal to adopt the sequence with vector reading frame. A study indicated that gly at the N-terminal of the peptide could promote target selectivity (36). The peptide was produced as a recombinant fusion protein in *E. coli* BL21, produced in inclusion bodies. Hence, it was solubilized with urea and purified on a chitin column after refolding. Dithiothreitol (DTT) was used as a reluctant agent to cleave the peptide from the fusion part at the intein site. The production yield was about 1 mg/L culture of recombinant host. 

MIC reported against *E. faecalis and S. aureus *for P24 was 64 µg/mL, which showed more potency than pexiganan (MIC 256 µg/mL). The present study demonstrated that P24 can be considered as an AMP against *S. aureus*. An old study reported the MIC of Pexiganan against *S. aureus, P. aeruginosa, E. coli, *and *K. pneumoniae* in the range of 8-16 µg/mL and against *E. faecalis, *64 µg/mL (43); however, our results in the triplicated analysis showed higher values for MIC of newly synthesized Pexiganan. It might be related to the quality of Pexiganan synthesis or the medium condition of the tests. Therefore, further tests using new standard samples should be performed in the future studies. However, in general term, P24 activity against *P. aeruginosa* was similar to pexiganan. P-24 was more effective against gram-positive bacteria than gram-negative. However, this AMP, P24, had a hemolytic effect, which was not seen by Pexiganan (35). This may be due to the highly positive charges of novel peptide. Dathe *et.al* demonstrated that increasing net charge up to +5 intensified antimicrobial activity and cell selectivity; however, promoting net charge more than five would decrease the therapeutic index of AMP (44). According to the findings, reducing the positive charge in P-24 might be a solution to reduce the hemolytic effect. 

**Table 1.** Physicochemical properties of P24.

**Name**	**No. of residue**	**Charge** **In pH 7**	**pI**	**Extinction coefficient**	**MW (g/mol)**	**Water Solubility**
P24	24	10.1	13.05	22760 M^-1^cm^-1^	3287.91	Good

**Table 2:** Antimicrobial activity of P24 compared to Pexiganan

***K. pneumonia*** **ATCC 13883**	***E. coli*** **ATCC 25922**	***E. faecalis*** **ATCC 29212**	***S. aureus*** **ATCC 25923**	***P*** **. ** ***aeruginosa*** **ATCC 27853**	**Bacteria**
**MIC (µg/mL)**	**MIC (µg/mL)**	**MIC (µg/mL)**	**MIC (µg/mL)**	**MIC (µg/mL)**	
512	512	256	256	256	Pexiganan
128	128	64	64	256	P24 (new AMP)

## References

[1] Oskay M, Oskay D, Kalyoncu F (2009). Activity of some plant extracts against multi-drug resistant human pathogens. Iran. J. Pharm. Res.

[2] Tehrani MM, Erfani M, Amirmozafari N, Nejadsattari T (2019). Synthesis of a peptide derivative of microcinJ25 and evaluation of antibacterial and biological activities. Iran. J. Pharm. Res.

[3] Mehrdar MT, Madani R, Hajihosseini R, Bidhendi SM (2017). Antibacterial activity of isolated immunodominant proteins of Naja Naja (Oxiana) Venom. Iran. J. Pharm. Res.

[4] Ong ZY, Wiradharma N, Yang YY (2014). Strategies employed in the design and optimization of synthetic antimicrobial peptide amphiphiles with enhanced therapeutic potentials. Adv. Drug Deliv. Rev.

[5] Deslouches B, Steckbeck JD, Craigo JK, Doi Y, Mietzner TA, Montelaro RC (2013). Rational design of engineered cationic antimicrobial peptides consisting exclusively of arginine and tryptophan, and their activity against multidrug-resistant pathogens. Antimicrob. Agents Chemother.

[6] Guralp SA, Murgha YE, Rouillard JM, Gulari E (2013). From design to screening: a new antimicrobial peptide discovery pipeline. PLoS One.

[7] Li Y, Xiang Q, Zhang Q, Huang Y, Su Z (2012). Overview on the recent study of antimicrobial peptides: origins, functions, relative mechanisms and application. Peptides.

[8] Haney EF, Mansour SC, Hancock REW, Hansen PR (2017). Antimicrobial Peptides: An Introduction. ntimicrobial Peptides: Methods and Protocols.

[9] Tam JP, Wang S, Wong KH, Tan WL (2015). Antimicrobial peptides from plants. Pharmaceuticals(Basel).

[10] Li Y (2011). Recombinant production of antimicrobial peptides in Escherichia coli: a review. Protein Expr. Purif.

[11] Zorko M, Jerala R, Giuliani A, Rinaldi A (2010). Production of recombinant antimicrobial peptides in bacteria. Antimicrobial Peptides. Methods in Molecular Biology (Methods and Protocols).

[12] Wanmakok M, Orrapin S, Intorasoot A, Intorasoot S (2018). Expression in Escherichia coli of novel recombinant hybrid antimicrobial peptide AL32-P113 with enhanced antimicrobial activity in-vitro. Gene.

[13] Shi C, Miskioglu EE, Meng Q, Wood DW (2013). Intein-based purification tags in recombinant protein production and new methods for controlling self-cleavage. Pharm. Bioprocess.

[14] Mitchell SF, Lorsch JR, Lorsch JR (2015). Chapter Eight - Protein Affinity Purification using Intein/Chitin Binding Protein Tags. Methods in Enzymology.

[15] Kosobokova E, Skrypnik K, Kosorukov V (2016). Overview of fusion tags for recombinant proteins. Biochem. (Mosc.).

[16] Chong S, Shao Y, Paulus H, Benner J, Perler FB, Xu MQ (1996). Protein splicing involving the Saccharomyces cerevisiae VMA intein: The steps in the splicing pathway, side reactions leading to protein cleavage, and establishment of an in-vitro splicing system. J. Biol. Chem.

[17] Fang YT, Lai WS, Liu JH, Liu YC (2019). Enhanced cecropin B2 production via chitin‐binding domain and intein self‐cleavage system. Biotechnol. Appl. Biochem.

[18] Hong I, Kim YS, Choi SG (2010). Simple purification of human antimicrobial peptide dermcidin (MDCD-1L) by intein-mediated expression in E. coli. J. Microbiol. Biotechnol.

[19] Chen YQ, Zhang SQ, Li BC, Qiu W, Jiao B, Zhang J, Diao ZY (2008). Expression of a cytotoxic cationic antibacterial peptide in Escherichia coli using two fusion partners. Protein Expr. Purif.

[20] Morassutti C, De Amicis F, Bandiera A, Marchetti S (2005). Expression of SMAP-29 cathelicidin-like peptide in bacterial cells by intein-mediated system. Protein Expr. Purif.

[21] Roudi R, Syn NL, Roudbary M (2017). Antimicrobial peptides as biologic and immunotherapeutic agents against cancer: A comprehensive overview. Front. Immunol.

[22] Wang G (2015). Improved methods for classification, prediction, and design of antimicrobial peptides. Computational Peptidology.

[23] Sharma A, Gupta P, Kumar R, Bhardwaj A (2016). DPABBs: A novel in-silico approach for predicting and designing anti-biofilm peptides. Sci. Rep.

[24] Jorgensen JH, Ferraro MJ (2009). Antimicrobial susceptibility testing: a review of general principles and contemporary practices. Clin. Infect. Dis.

[25] Kim H, Jang JH, Kim SC, Cho JH (2014). De novo generation of short antimicrobial peptides with enhanced stability and cell specificity. J. Antimicrob. Chemother.

[26] Carretero GPB, Saraiva GKV, Cauz ACG, Rodrigues MA, Kiyota S, Riske KA, dos Santos AA, Pinatto-Botelho MF, Bemquerer MP, Gueiros-Filho FJ, Chaimovich H, Schreier S, Cuccovia IM (2018). Synthesis, biophysical and functional studies of two BP100 analogues modified by a hydrophobic chain and a cyclic peptide. Biochim. Biophys. Acta Biomembr.

[27] Ferre R, Melo MN, Correia AD, Feliu L, Bardaji E, Planas M, Castanho M (2009). Synergistic effects of the membrane actions of cecropin-melittin antimicrobial hybrid peptide BP100. Biophys. J.

[28] Shen Y, Maupetit J, Derreumaux P, Tufféry P (2014). Improved PEP-FOLD approach for peptide and miniprotein structure prediction. J. Chem. Theory Comput.

[29] Zasloff M, Martin B, Chen HC (1988). Antimicrobial activity of synthetic magainin peptides and several analogues. Proc. Natl. Acad. Sci.

[30] Rodríguez A, Villegas E, Montoya-Rosales A, Rivas-Santiago B, Corzo G (2014). Characterization of antibacterial and hemolytic activity of synthetic pandinin 2 variants and their inhibition against Mycobacterium tuberculosis. PLoS One.

[31] Pedron CN, Torres MDT, da Silva Lima JA, Silva PI, Silva FD, Oliveira VX (2017). Novel designed VmCT1 analogs with increased antimicrobial activity. Eur. J. Med. Chem.

[32] Juretić D, Vukičević D, Tossi A, Hansen PR (2017). Tools for designing amphipathic helical antimicrobial peptides. Antimicrobial Peptides: Methods and Protocols.

[33] Szweda P, Pladzyk R, Kotlowski R, Kur J (2001). Cloning, expression, and purification of the Staphylococcus simulans lysostaphin using the intein-chitin-binding domain (CBD) system. Protein Expr. Purif.

[34] Wu C, Seitz PK, Falzon M (2000). Single-column purification and bio-characterization of recombinant human parathyroid hormone-related protein (1-139). Mol. Cell. Endocrinol.

[35] Gottler LM, Ramamoorthy A (2009). Structure, membrane orientation, mechanism, and function of pexiganan—a highly potent antimicrobial peptide designed from magainin. Biochim. Biophys. Acta Biomembr.

[36] Le CF, Yusof MYM, Hassan H, Sekaran SD (2015). In-vitro properties of designed antimicrobial peptides that exhibit potent antipneumococcal activity and produces synergism in combination with penicillin. Sci. Rep.

[37] Pathak N, Salas‐Auvert R, Ruche G, Janna MH, McCarthy D, Harrison RG (1995). Comparison of the effects of hydrophobicity, amphiphilicity, and α‐helicity on the activities of antimicrobial peptides. Proteins.

[38] Zhao J, Zhao C, Liang G, Zhang M, Zheng J (2013). Engineering antimicrobial peptides with improved antimicrobial and hemolytic activities. J. Chem. Inf. Model.

[39] Kim H, Jang JH, Kim SC, Cho JH (2013). De novo generation of short antimicrobial peptides with enhanced stability and cell specificity. J. Antimicrob. Chemother.

[40] Banki MR, Gerngross TU, Wood DW (2005). Novel and economical purification of recombinant proteins: intein‐mediated protein purification using in-vivo polyhydroxybutyrate (PHB) matrix association. Protein Sci.

[41] Lu W, Sun Z, Tang Y, Chen J, Tang F, Zhang J, Liu JN (2011). Split intein facilitated tag affinity purification for recombinant proteins with controllable tag removal by inducible auto-cleavage. J. Chromatogr. A.

[42] Elleuche S, Pöggeler S (2010). Inteins, valuable genetic elements in molecular biology and biotechnology. Appl. Microbiol. Biotechnol.

[43] Ge Y, MacDonald DL, Holroyd KJ, Thornsberry C, Wexler H, Zasloff M (1999). In-vitro antibacterial properties of pexiganan, an analog of magainin. Antimicrob. Agents Chemother.

[44] Oddo A, Hansen PR, Hansen PR (2017). Hemolytic Activity of Antimicrobial Peptides. Antimicrobial Peptides: Methods and Protocols.

